# The pathogenicity of genetic variants previously associated with left ventricular non‐compaction

**DOI:** 10.1002/mgg3.182

**Published:** 2015-12-20

**Authors:** Yeganeh Abbasi, Javad Jabbari, Reza Jabbari, Ren‐Qiang Yang, Bjarke Risgaard, Lars Køber, Stig Haunsø, Jacob Tfelt‐Hansen

**Affiliations:** ^1^ The Danish National Research Foundation Center for Cardiac Arrhythmia (DARC)CopenhagenDenmark; ^2^ Laboratory of Molecular CardiologyDepartment of CardiologyThe Heart CentreCopenhagen University Hospital RigshospitaletCopenhagenDenmark; ^3^ Department of CardiologyThe Heart CenterRigshospitaletCopenhagenDenmark; ^4^ Department of CardiologyInstitute of Cardiovascular DiseaseThe Heart CenterThe Second Affiliated HospitalNanchang UniversityNanchangChina; ^5^ Department of Clinical MedicineFaculty of Health and Medical ScienceUniversity of CopenhagenCopenhagenDenmark

**Keywords:** Exome aggregation consortium, Exome sequencing project, genes, human gene mutation database, left ventricular non‐compaction, LVNC, LVNC‐associated variants, variants

## Abstract

**Background:**

Left ventricular non‐compaction (LVNC) is a rare cardiomyopathy. Many genetic variants have been associated with LVNC. However, the number of the previous LVNC‐associated variants that are common in the background population remains unknown. The aim of this study was to provide an updated list of previously reported LVNC‐associated variants with biologic description and investigate the prevalence of LVNC variants in healthy general population to find false‐positive LVNC‐associated variants.

**Methods and Results:**

The Human Gene Mutation Database and PubMed were systematically searched to identify all previously reported LVNC‐associated variants. Thereafter, the Exome Sequencing Project (ESP) and the Exome Aggregation Consortium (ExAC), that both represent the background population, was searched for all variants. Four in silico prediction tools were assessed to determine the functional effects of these variants. The prediction results of those identified in the ESP and ExAC and those not identified in the ESP and ExAC were compared. In 12 genes, 60 LVNC‐associated missense/nonsense variants were identified. *MYH7* was the predominant gene, encompassing 24 of the 60 LVNC‐associated variants. The ESP only harbored nine and ExAC harbored 18 of the 60 LVNC‐associated variants. In total, eight out of nine ESP‐positive variants overlapped with the 18 variants identified in ExAC database.

**Conclusions:**

In this article, we identified 9 ESP‐positive and 18 ExAC‐positive variants of 60 previously reported LVNC‐associated variants, suggesting that these variants are not necessarily the monogenic cause of LVNC.

## Introduction

Left ventricular non‐compaction (LVNC) is a rare cardiomyopathy with a prevalence that varies considerably among studies (0.014–14%) (Oechslin et al. 2000; Pignatelli et al. 2003; Stöllberger and Finsterer 2005; Aras et al. 2006; Belanger et al. 2008; Stanton et al. 2009; Tian et al. 2014). Left ventricular non‐compaction is most likely caused by a pathological arrest in the compaction process, which leads to a non‐compacted left ventricular myocardium (Dusek et al. 1975; Chin et al. 1990; Agmon et al. 1999; Zambrano et al. 2002). Left ventricular non‐compaction is characterized by a non‐compacted inner myocardial layer and a compacted outer myocardial layer with deep intratrabecular recesses, that are communicated to the left ventricular chamber (Jenni et al. 2001; Sarma et al. 2010).

There are no single diagnostic criteria for LVNC. The clinical presentation is highly variable from asymptomatic to symptomatic with heart failure (HF), atrial and ventricular arrhythmias, thromboembolic events, and sudden cardiac death (Ichida et al. 1999; Paterick et al. 2010; Bhatia et al. 2011). Left ventricular non‐compaction is associated with congenital cardiac disorders, other cardiomyopathies, and some neuromuscular diseases (Jenni et al. 2001).

Both sporadic and familial LVNC have been reported (Sasse‐Klaassen et al. 2003; Sen‐Chowdhry and McKenna 2008). The pattern of familial LVNC inheritance is mainly autosomal dominant with incomplete penetrance, although autosomal recessive and X‐linked inheritance have been reported (Bleyl et al. 1997a; Sasse‐Klaassen et al. 2003; Xing et al. 2006; Sen‐Chowdhry and McKenna 2008). Left ventricular non‐compaction is associated with variants in mitochondrial, cytoskeletal, Z‐line, and sarcomeric genes (Sen‐Chowdhry and McKenna 2008). The sarcomeric genes include cardiac *β*‐myosin heavy chain (*MYH7*), cardiac troponin T (*TNNT2*), and cardiac *α*‐actin (*ACTC1*) (Budde et al. 2007; Hoedemaekers et al. 2007; Monserrat et al. 2007; Klaassen et al. 2008). Furthermore, LVNC has been described with variants in tafazzin (*TAZ*), alpha‐dystrobrevin (*DTNA*), tropomyosin 1 (*TPM1*), and cardiac troponin I (*TNNI3*) (Ichida et al. 2001; Budde et al. 2007; Klaassen et al. 2008; Probst et al. 2011; Arndt et al. 2013). Carriers of these sarcomeric variants demonstrate a range of phenotypes from no LVNC (asymptomatic) to early onset LVNC or other cardiomyopathies (Moric‐Janiszewska and Markiewicz‐Łoskot 2008; Finsterer 2009).

The first gene associated with LVNC, tafazzin, was reported more than 17 years ago (Bleyl et al. 1997a). Ongoing genetic research has since discovered many novel variants in the genes described above. Despite the known associated genes, the genetic background of this cardiomyopathy is not fully understood.

The aim of this article was to provide an updated list of LVNC‐associated variants previously reported in the literatures with biologic description. In addition, we will investigate the prevalence of LVNC variants in a healthy general population in order to identify false‐positive LVNC‐associated variants.

## Methods

### Data collection

Genes and variants previously associated with LVNC were identified using the Human Gene Mutation Database (HGMD) and by reviewing published literature in PubMed until July 17th 2015 (HGMD (Human Gene Mutation Database for Human Genetics Research); Exome Variant Server).

The following queries were used: ((Left ventricular non‐compaction), (Left ventricular noncompaction), (left ventricular hypertrabeculation) and (left ventricular hypertrabecular/non‐compaction [Mesh])) and ((Genetic) or (“Genetics” [Mesh])), in order to collect all published data on identified LVNC‐associated variants. Furthermore, we also searched HGMD, Google scholar, and publicly available databases.

All relevant articles were reviewed for data on functional studies and on familial co‐segregation. We defined co‐segregation as at least two genotypically positive family members with the same phenotype. Positive functional data were determined as any in vivo or in vitro model demonstrating results differing from the wild‐type model as described previously.

### Exome sequencing project

In the Exome Sequencing Project (ESP), next‐generation sequencing of all protein coding regions in 6503 individuals of African American (*n* = 2203 individuals) and European American (*n* = 4300 individuals) descent from different population studies has been carried out. Clinical data were not readily available or available on request.

The ESP database was searched for the missense and nonsense variants previously associated with LVNC.

Left ventricular non‐compaction‐associated variants were subdivided into those that were identified in the ESP database (ESP positive) and those that were not identified in the ESP database (ESP negative).

### The exome aggregation consortium

In the exome aggregation consortium, sequenced 60,706 unrelated individuals on the regions of the human genome that encode proteins as part of various disease‐specific and population genetic studies. Individuals with sever pediatric disease have been removed. Thus, the ExAC Browser is a very useful dataset of allele frequencies. Left ventricular non‐compaction‐associated variants were subdivided into those that were identified in the ExAC database (ExAC positive) and those that were not identified in the ExAC database (ExAC negative).

### Prediction analysis

The functional effects of all variants were assessed using four prediction tools: (1) Conservation across species; (2) Grantham Score; (3) Sorting Intolerant From Tolerant, v5.1.1 (SIFT); and (4) PolyPhen‐2. Conservation across species was obtained from the HGMD and classified as occurring at a position with no substitutions (conserved/pathogenic) or ≥1 substitutions (not conserved/benign). Grantham physicochemical values were calculated using the Grantham amino acid difference matrix. We defined a value above 100 as radical (pathogenic) and a value under 100 as conservative (benign) (Grantham 1974; Giudicessi et al. 2012). Using PolyPhen‐2, each variant was labeled as “probably damaging,” “possibly damaging,” or “benign” (PolyPhen‐2). Variants labeled “probably damaging” and “possibly damaging” were considered to be “damaging” (pathogenic) in our analysis. Finally, SIFT prediction classified variants as “tolerant” (benign) or “damaging” (pathologic) (SIFT).

In a final analysis using all prediction tools, a variant was considered damaging (pathogenic) if ≥3 in silico prediction tools identified the variant as pathogenic, as previously described (Giudicessi et al. 2012; Andreasen et al. 2013; Jabbari et al. 2013; Risgaard et al. 2013). Variants predicted to be damaging by one or two tools were considered to be variants of uncertain significance (VUS). Each of the four prediction tools predicted separately the pathogenicity of previously published LVNC‐associated variants. We used Giudicessi et al. (2012) agreement of ≥3 in silico tool predictions to assess the pathogenic status of a LVNC‐associated variant. This is an established and robust bioinformatics method, increasing the importance of the assessment using the known online prediction tools. (Giudicessi et al. 2012; Andreasen et al. 2013; Jabbari et al. 2013; Risgaard et al. 2013) This method increases the correlation between genetic findings and clinical practice especially in rare diseases.

**Figure 1:**
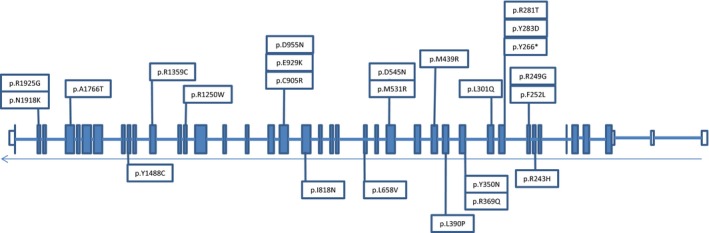
LVNC‐associated variants in the *MYH7* exons. White exons are untranslated region and blue exons are translated sequence.

## Results

Through a review of the literature, we identified 12 genes previously associated with LVNC (Table S1). Overall, we identified 60 missense/nonsense variants previously associated with LVNC, and most of the variants (40%) were found in *MYH7* (24 out of 60).

The ESP database included 9 of these 60 variants, which were found in seven genes: *MYBPC3, MYH7B, MIB, TNNT2, CASQ2, TAZ*, and *LDB3* (Table S1). Notably, no variants in *MYH7* were found in the ESP database. In the ExAC database, we identified 18 variants distributed in all genes except *TPM1*. In total, eight out of nine ESP‐positive variants overlapped with the 18 variants identified in ExAC database (Table S1).

Two variants (p.D545N and p.D955N) coexisted as a heterozygous double mutation in the cis position on the same *MYH7* allele segregating with LVNC. Notably, the patients were of various ethnic backgrounds and nationalities from Europe, America, and Asia. Most of the patients were men, and the patient age spectrum was widespread from infancy to adulthood.

### Heredity pattern

In total, we found 42 familial forms of LVNC. Familial genetic co‐segregation was found in 30 out of 42 clinical familial cases (Table S1). Some of these variants were also identified in other studies not having a clear heredity pattern, suggesting a sporadic form of LVNC. Therefore, there is some overlap between familial and sporadic cases.

In the familial cases, three heredity patterns, including autosomal dominant, autosomal recessive, and X‐linked, were reported. The majority of the heredity patterns were autosomal dominant. We found only one example of autosomal recessive inheritance pattern among all familial cases (p.R820W/*MYBPC3*). Some variants showed both sporadic and familial inheritance patterns in different studies (p.G197R/*TAZ*) (Bleyl et al. 1997b).

### Phenotypes of the families

We found a wide clinical spectrum of LVNC phenotypes ranging from asymptomatic to symptomatic (Table S1). Left ventricular non‐compaction was the predominant form of cardiomyopathy in the families (39 of 41). However, in some families, the relatives showed other cardiomyopathies (dilated cardiomyopathy (DCM) and hypertrophic cardiomyopathy (HCM)) with no sign of LVNC (Table S1). There were also four families with co‐existing LVNC and DCM, and two families showed LVNC and HCM, and one family exhibited LVNC and a congenital heart defect (Ichida et al. 2001). Notably, we found two cases with several types of cardiac diseases in their families (Postma et al. 2011; Ronvelia et al. 2012). In a family with the *TAZ*/p.G195* variant, LVNC co‐existed with Barth syndrome, DCM, atrial septal defect, and elevated urine 3‐methylglutaconic acid (3‐MGA) levels (Ronvelia et al. 2012). Lastly, LVNC co‐existed with cardiovascular malformation and Ebstein᾿s anomaly in a family with the *MYH7/*p.Y283D variant (Postma et al. 2011). These results highlighted that multiple factors affect the pathogenicity of variants, but family‐based whole‐genome sequencing makes it possible to identify the association between a genetic variant and a rare disease.

### Prediction analysis

Prediction analysis was carried out for 56 of the 60 variants. The remaining four variants were not analyzed because they generated stop codons.

By utilizing the Giudicessi et al. (2012) agreement of ≥3 in silico tools, prediction analysis determined that 51% of missense variants were pathogenic, 41% were VUS, and 5% were benign. The pathogenicity of the p.D626N/*LDB3* variant could not be predicted using the agreement of ≥3 in silico tools and it did not match with the amino acids in six isoforms of the protein.

Finally, by using the Giudicessi et al. (2012) agreement of ≥3 in silico tools, 44% (4/9) of the ESP‐positive variants were pathogenic compared with 55% (10/18) of the ExAC‐positive variants (Table S1).

## Discussion

Using bioinformatics, this is the first study to identify and investigate, all previous genetic variants associated with LVNC until July 2015. We found 60 variants in 12 genes associated with LVNC. In addition, prediction analyses were carried out for all missense variants.

This study identified 9 LVNC‐associated variants in the ESP database and 18 variants in ExAC database.

In the *MYH7* gene, 24 of 60 LVNC‐associated variants were reported. None of those were identified in the ESP, but two SNPs (p.R1359C and p.R243H) were identified in ExAC database (Klaassen et al. 2008). These two variants predicted to be pathogenic with agreement of more than three prediction tools analysis. The assumption is that a pathogenic variant is unlikely to be present in a high number of individuals in the background population. The cut‐off for how many persons, one variant can be present in a cohort like ESP and still be pathogenic, is still unclear. Also, it should be kept in mind that it is of course possible that the geno‐positive individuals in general population can develop LVNC or other cardiomyopathies in late age. The p.R1359C variant in *MYH7* was a sporadic case of LVNC, while the p.R243H was identified with family co‐segregation and with low allele frequency (0.000008236) in the ExAC database (Exome Aggregation Consortium (ExAC)). These findings suggest that the p.R243H theoretically develop LVNC in healthy carriers. Family co‐segregation is a valuable tool for determining the pathogenicity of disease‐causing variants (Klaassen et al. 2008). Hoedemaekers et al. (2010) performed a family screening with both molecular and clinical examinations in 58 patients with LVNC and their 194 family members. This screening revealed that 67% (39/58 patients with LVNC) had a genetic background with these variants. Furthermore, nine of 50 families had one of these variants. The findings support that the variants in *MYH7* penetrate to the next generation. Budde et al. (2007) assessed the clinical and molecular characterization of a large German family with LVNC. The variant p.R281T in *MYH7* was present in affected family members (11/24) and also the p.R281T known as ESP‐negative and ExAC‐negative variant. These results supported the major role of *MYH7* variants in the development of LVNC.

Kaneda et al. (2008) screened 99 unrelated probands with DCM and five probands with isolated LVNC and reported a novel p.M531R variant in *MYH7* in a patient with isolated LVNC, and we did not find the p.M531R in ESP and ExAC databases. To define the function of the p.M531R variant in *MYH7*, Kaneda et al. (2008) performed a functional study with transgenic mouse carrying the p.M532R mutant alpha MHC gene, which is identical to the p.M531R variant in human beta MHC. Although none of the mice had the characteristics of LVNC, 50–70% of the transgenic mice demonstrated left ventricular hypertrophy at 2–3 months of age. In addition, dilation of the left ventricle was reported in approximately 25% of transgenic mice by 18 months of age. They showed biphasic changes in the LV wall thickness (HCM developing to DCM). Their results suggested that the p.M531R variant has a malignant or pro cardiomyopathic effect on cardiac function.

By using four prediction analysis, 17 out of 24 (71%) previously reported LVNC‐associated *MHY7* variants were predicted pathogenic, and interestingly, none of variants in *MYH7* were predicted benign. Due to the effect of LVNC‐associated variants in *MYH7* on cardiac development and function, the genetic basis of this gene in LVNC appears to be important. The variants found in *MYH7* in patients with isolated LVNC strongly support the importance of this gene in pathogenesis of LVNC. Functional investigations of these variants are important and needed to provide a more comprehensive understanding of the effects of variants on protein function.

We identified LVNC‐associated genes (*DTNA*,* MYH7B* and *CASQ2*) that had only one nonsynonym LVNC‐associated variant (p.P121L, p.R890C, and p.H244R) (Ichida et al. 2001; Hoedemaekers et al. 2010; Esposito et al. 2013). The p.H244R and p.R890C variants present in both ESP and ExAC database, and the p.P121L variant only present in ExAC database. In all of these variants, there was family co‐segregation with the same phenotype (LVNC) (Ichida et al. 2001; Hoedemaekers et al. 2010; Esposito et al. 2013). The genetic screening in the first degree relatives to identify co‐segregation is an important tool to determine the pathogenicity of a variant. These co‐segregation data suggest that these variants are likely to be disease causing in a monogenic pattern, while they are predicted benign, VUS, and pathogenic (Table S1).

*MYBPC3* contains seven LVNC‐associated variants, of which three (p.G5R, p.G490R and p.R502W) are found in the ESP database and six variants (p.R820W, p.P873L, p.G5R, p.G490R, p.G148R, and p.R502W) identified in ExAC Browser (Table S1). Interestingly, p.R820W exhibited autosomal recessive inheritance, whereas the rest displayed autosomal dominant inheritance. The p.G490R and the p.R502W predicted as pathogenic variants, while they are present in both ESP database and ExAC database. These variants were also identified in a family co‐segregation study. The family members with the p.R502W developed HCM and the family member with the p.G490R developed LVNC. These data point to the fact that there are many factors (for example modifier genes, age, environment, etc.) that affect the pathogenicity of variants.

Functional studies are often helpful to clarify the function of the protein variants such as rare and low‐frequency LVNC‐associated variants. Functional studies have been performed for eight LVNC‐associated variants (*MYH7*/p.M531R, *MIB1*/p.R530*, and p.V943F, *PRDM16*/p.K702*, *TNNT2*/p.E96K ,and p.R131W, *LDB3*/p.D117N, and *TPM1*/p.D84N). All variants showed loss‐of‐function in mutated proteins (Table S1).

Accurate estimating of rare variants frequency in background population is needed with a widespread ethnic background population with enormous number of healthy individuals. Multiple factors (e.g., modifier genes, age, environment, etc.) affect the penetrance of pathogenic variants in healthy individuals. Thus, it is not possible to determine the possibility of pathogenicity for ExAC‐positive variant. Additional functional study and genetic searching in family members are needed to clarify the pathogenic effect of these ExAC‐positive variants in general population.

### Limitations

One limitation of this study is that the variants reported in this manuscript were collected in patients of different nationalities worldwide, while the ESP is restricted to European American and African American patients. Thus, the prevalence of LVNC in individual carrying LVNC‐associated variants in the ESP and ExAC could not been determined.

Due to lack of data regarding variants positioned in promoters, introns, and untranslated regions (UTR) in the ESP, variants found in these regions were not included in our study. The clinical status of variant carriers was not readily available. It is possible that some of individuals suffer from LVNC or other cardiomyopathies in the ExAC population.

Functional investigations have only been carried out for a minority of the variants, and not all studies investigated whether a variant co‐segregated.

### Implications

This novel dataset should be used in the evaluation of potential disease‐causing variants. The presence of a potential disease‐causing variant in these large databases implies that the variant is less likely to be disease causing. The presence of a variant in one person in the large databases (more than 5000 persons) does not rule out disease causality, but in our opinion, the presence of a variant in more than five persons makes it unlikely to be pathogenic.

## Conclusions

In this analysis, we demonstrate that *MYH7* is the predominant gene associated with LVNC. *MYH7* variants comprised 24 of the total 60 variants. None of these variants were found in the ESP, but two of the variants were found in the ExAC database. Family members showed a widespread clinical spectrum from asymptomatic to symptomatic forms of LVNC and other cardiomyopathies.

We also identified nine ESP‐positive and 18 ExAC‐positive variants of 60 previously reported LVNC‐associated variants, suggesting that these variants are not necessarily the monogenic cause of LVNC. Thus, additional data from functional studies and genetic screening in family members is still important in order to help clinician to estimate whether a variant is truly disease causing or not.

## Conflict of Interest

Javad Jabbari is employed at LEO Pharma A/S. There are no financial interests to report.

## Supporting information


**Table S1.** LVNC‐associated genes and variants.
